# Credibility of subgroup analyses by socioeconomic status in public health intervention evaluations: An underappreciated problem?

**DOI:** 10.1016/j.ssmph.2018.09.010

**Published:** 2018-10-19

**Authors:** Greig Inglis, Daryll Archibald, Lawrence Doi, Yvonne Laird, Stephen Malden, Louise Marryat, John McAteer, Jan Pringle, John Frank

**Affiliations:** Scottish Collaboration for Public Health Research and Policy, Usher Institute of Population Health Sciences and Informatics, University of Edinburgh, 20 West Richmond Street, EH8 9DX, United Kingdom

**Keywords:** Health inequalities, Health inequities, Equity and public health interventions, Policy impact by socioeconomic status

## Abstract

There is increasing interest amongst researchers and policy makers in identifying the effect of public health interventions on health inequalities by socioeconomic status (SES). This issue is typically addressed in evaluation studies through subgroup analyses, where researchers test whether the effect of an intervention differs according to the socioeconomic status of participants. The credibility of such analyses is therefore crucial when making judgements about how an intervention is likely to affect health inequalities, although this issue appears to be rarely considered within public health. The aim of this study was therefore to assess the credibility of subgroup analyses in published evaluations of public health interventions. An established set of 10 credibility criteria for subgroup analyses was applied to a purposively sampled set of 21 evaluation studies, the majority of which focussed on healthy eating interventions, which reported differential intervention effects by SES. While the majority of these studies were found to be otherwise of relatively high quality methodologically, only 8 of the 21 studies met at least 6 of the 10 credibility criteria for subgroup analysis. These findings suggest that the credibility of subgroup analyses conducted within evaluations of public health interventions’ impact on health inequalities may be an underappreciated problem.

## 1 Introduction

There is a clear social gradient in the vast majority of health outcomes, whereby morbidity and premature mortality are concentrated amongst the most socioeconomically deprived groups in society. Health inequalities by socioeconomic status (SES) are caused by a combination of bio-psycho-social exposures acting over the life course (Hertzman et al., 1994, Hertzman and Boyce, 2010), and these exposures are themselves patterned by unequal distributions of power, wealth and income across society (Marmot, Friel, Bell, Houweling, & Taylor, 2008).

Reducing health inequalities between the most and least socioeconomically deprived groups in society has been identified as a priority for policymakers in the UK for nearly four decades, although little progress has been made in reducing these inequalities to date (Bleich et al., 2012, Frank and Haw, 2011, Frank and Haw, 2013, Mackenbach, 2011, McCartney et al., 2017). Within this context, there is increasing interest in identifying promising public health interventions, or social policies, which may be effective in reducing health inequalities, by achieving differentially large health gains in the most socioeconomically deprived groups in society. Similarly, there is a growing recognition, and concern, that some interventions or policies may increase health inequalities if they disproportionately benefit the most affluent groups in society, an effect termed “intervention generated inequalities” (Lorenc, Petticrew, Welch, & Tugwell, 2012). There now exists a large body of literature on the potential differential effects of a wide variety of public health interventions and policies, across a number of different target outcomes and levels of action, many of which have been summarised in reviews (Hill et al., 2014, Hillier-Brown et al., 2014, McGill et al., 2015) and “umbrella” reviews of reviews (Bambra et al., 2010, Lorenc et al., 2012). We became aware of the issue of subgroup analysis credibility whilst reviewing the main approaches that have been taken for the classification of public health interventions:the “sector” approach of Bambra et al. (2010); the “six Ps” approach of McGill et al. (2015); and the “degree of individual agency” approach of Adams, Mytton, White, and Monsivais (2016). In the course of this work, we became aware of recurring methodological issues with the subgroup analyses reported. We therefore decided to examine more closely the methodological quality of widely cited and high-quality-rated public health intervention studies, claiming to demonstrate differential effects by social class. We report those findings here.

Clearly, an important issue for consumers of evaluation research to consider is the “credibility” of such analyses: the extent to which a putative subgroup effect can confidently be asserted to be believable or real (Sun, Briel, Walter, & Guyatt, 2010). Clinical epidemiological methodologists have proposed guidelines for conducting credible subgroup analyses within randomised control trials (RCTs) and assessing the credibility of reported subgroup effects, although this guidance may not always be applied by researchers in practice. For example, recent systematic reviews clinical trials in the medical literature (Sun et al., 2012) and back pain specifically (Saragiotto et al., 2016) have shown that the majority of apparent subgroup effects that are reported do not meet many of the established criteria for credible subgroup analyses (Burke, Sussman, Kent & Hayward, 2015; Oxman & Guyatt, 1992; Sun et al., 2010). Both of these reviews examined differential effects according to a range of population subgroups beyond those defined by SES, such as those defined by age and gender.

There has to date been relatively little discussion of subgroup analysis credibility in evaluations of public health interventions generally, or with respect to health inequalities by SES specifically. This is an important issue given the role of such analyses in guiding decision making, regarding interventions that may reduce health inequalities: high quality, credible subgroup analyses can shed light on how interventions may either reduce or increase health inequalities and are therefore invaluable to aid effective decision making. Non-credible subgroup analyses, on the other hand, may produce spurious differential intervention effects by SES and lead to decision makers drawing erroneous conclusions about the effects of an intervention on health inequalities. In this paper, we reflect on our experience of assessing the credibility of subgroup analyses in a purposive sample of public health primary intervention evaluation studies that report differential impacts by SES.

## 2 Methods

We aimed to purposively sample a diverse set of evaluations of public health interventions that reported differential health impacts by any marker of SES. [By “purposive,” we mean a sampling strategy which stopped once we had identified a set of methodological issues, related to subgroup analyses in such studies, which seemed not to be augmented by including further studies – i.e. the yield of insights obtained for the reviewing effort expended was clearly reaching a plateau.] Specifically, we aimed to identify a pool of intervention studies, that had been already quality-appraised in at least one recent structured review, and which claimed to show an impact on health inequalities by SES. We wanted a sample of studies that were sufficiently diverse, in terms of the sorts of interventions evaluated and settings studied, so as to provide good coverage across all three of the published categorisation systems for such interventions (see above), in case those might be correlated with generaliseability of study findings. Our inclusion criteria were therefore that studies had to: i) be critically appraised as being of “moderate” to “high” quality in a structured review published in the last decade; ii) report on the evaluation of a public health intervention - meaning programmes or policies delivered at a higher level of aggregation than individual patients; iii) describe a public health intervention that was applicable to high-income countries; iv) evaluate the impact of a public health intervention with a credible study design and analysis (not limited to RCTs, to allow the inclusion of natural experiments and quasi-experimental designs (Craig et al., 2011)); v) report a differential effect of the intervention by SES.

We excluded studies that looked for a differential intervention effect by any marker of SES, such as income/family budget, education, or local-area average levels of deprivation, but did not find one (e.g. Nederkoorn, Havermans, Giesen, & Jansen, 2011). This decision was based on the fact that all but a handful the 21 studies we reviewed, which reported a differential effect by SES, utilised regression-based analyses with interaction (cross-product) terms for each interaction tested, between the observed intervention main effect, and the SES variable in question. We were well aware that such interaction analyses are notoriously low-powered (Brookes et al., 2004) but that the public health intervention literature rarely ever reports on the power of such analyses, even when none of the interactions examined are statistically significant, and the sample size of the study is unlikely to have been adequate for such interaction analyses. For example, the evaluation of altered food pricing by Nederkoorn et al., had only 306 subjects, half of whom were randomized to an online simulated food taxation intervention, but only 27% of whom had “low” daily food budgets – the SES marker examined. We refer the reader to more sophisticated guidance from academic disciplines, such as political science, which have long tended to have a more statistically sophisticated understanding of interaction analyses than the public health intervention literature (Brambor, Clark, & Golder, 2006).

Intervention studies meeting these criteria were located by reviewing the primary studies included in: i) McGill et al. (2015)'s review of socioeconomic inequalities in impacts of healthy eating interventions, where we selected those intervention studies that the authors had identified as being likely to reduce or increase health inequalities by preferentially improving healthy eating outcomes among lower and higher SES participants respectively, and that the authors had also assigned a quality score of 3 or greater; and ii) Bambra et al. (2010)'s umbrella review of interventions designed to address the social determinants of health (which yielded a further 3 primary studies). We reasoned that these reviews would provide a suitably diverse sample of primary studies as these were the sources where we had originally identified the Six Ps and Sectoral approaches to categorising interventions. An additional two recent studies (Batis et al., 2016, Colchero et al., 2016) that were previously known to us, were also included in order to include evaluations of societal-level policies through natural experiments. The final number of primary studies was 21 (See Table 1).

**Table 1:** Credibility criteria for credible subgroup analyses.

Subgroup analysis credibility criteria	Description (from Saragiotto et al., 2016)
Is the subgroup variable a characteristic measured at baseline?	Subgroup variables measured after randomisation might be influenced by the tested interventions. The apparent difference of treatment effect between subgroups can be explained by the intervention, or by differing prognostic characteristics in subgroups that appear after randomisation.
Was the subgroup variable a stratification factor at randomisation?	Credibility of subgroup difference would be increased if a subgroup variable was also used for stratification at randomisation (i.e. stratified randomisation).
Was the hypothesis specified a priori?	A subgroup analysis might be clearly planned before to test a hypothesis. This must be mentioned on the study protocol (registered or published) or primary trial, when appropriate. Post-hoc analyses are more susceptible to bias as well as spurious results and they should be viewed as hypothesis generating rather than hypothesis testing.
Was the subgroup analysis one of a small number of subgroup analyses tested (≤5)?	The greater the number of hypotheses tested, the greater the number of interactions that will be discovered by chance, that is, the more likely it is to make a type I error (reject one of the null hypotheses even if all are actually true). A more appropriate analysis would account for the number of subgroups.
Was the test of interaction significant (interaction *p* < 0.05)?	Statistical tests of significance must be used to assess the likelihood that a given interaction might have arisen due to chance alone (the lower a P value is, the less likely it is that the interaction can be explained by chance).
Was the significant interaction effect independent, if there were multiple significant interactions?	When testing multiple hypotheses in a single study, the analyses might yield more than one apparently significant interaction. These significant interactions might, however, be associated with each other, and thus explained by a common factor.
Was the direction of the subgroup effect correctly pre-specified?	A subgroup effect consistent with the pre-specified direction will increase the credibility of a subgroup analysis. Failure to specify the direction or even getting the wrong direction weakens the case for a real underlying subgroup effect
Was the subgroup effect consistent with evidence from previous studies?	A hypothesis concerning differential response in a subgroup of patients may be generated by examination of data from a single study. The interaction becomes far more credible if it is also found in other similar studies. The extent to which a comprehensive scientific overview of the relevant literature finds an interaction to be consistently present is probably the best single index as to whether it should be believed. In other words, the replication of an interaction in independent, unbiased studies provides strong support for its believability.
Was the subgroup effect consistent across related outcomes?	The subgroup effect is more likely to be real if its effect manifest across all closely related outcomes. Studies must determine whether the subgroup effect existed among related outcomes.
Was there indirect evidence to support the apparent subgroups effect (biological rationale, laboratory tests, animal studies)?	We are generally more ready to believe a hypothesised interaction if indirect evidence makes the interaction more plausible. That is, to the extent that a hypothesis is consistent with our current understanding of the biologic mechanisms of disease, we are more likely to believe it. Such understanding comes from three types of indirect evidence: (i) from studies of different populations (including animal studies); (ii) from observations of interactions for similar interventions; and (iii) from results of studies of other related outcomes.

The credibility of the subgroup analyses reported within each of the studies was assessed against the ten criteria outlined by Sun et al. (2012). The criteria refer to various aspects of study design, analysis and context and were derived largely from the guidance originally produced originally by Oxman and Guyatt (1992), that was subsequently updated by Sun et al. (2010). Each study was assessed on these ten criteria using the scoring tool developed by Saragiotto et al. (2016), which allocates one scoring point for each of the criteria met, for a maximum score of ten. The ten criteria for credible subgroup analysis are outlined in Table 1, alongside Saragiotto et al. (2016)'s description of each.

Each study was also scored on the Effective Public Health Practice Project (EPHPP) Quality Assessment Tool (Thomas, Ciliska, Dobbins, & Micucci, 2004), in order to assess the overall methodological quality of the studies. The EPHPP is a time-honoured critical appraisal tool for public health intervention evaluations that can be applied to both randomised and non-randomised intervention evaluation studies, and is comprised of six domains: selection bias, design, confounders, blinding, data collection methods and withdrawals and dropouts. Each component is rated as either strong, moderate or weak according to a standardised scoring guide and these scores are subsequently summed to provide an overall quality score. Studies are rated as being strong overall if no components receive a weak score, moderate if one component recieves a weak rating and weak if two or more components receive a weak score.

Each of the 21 studies in our sample was rated according to the subgroup credibility criteria and also EPHPP by one of three pairs of reviewers. Each reviewer read and scored the studies independently, before meeting to discuss their scores and resolve any discrepancies in how each study had been rated.

## 3 Results

A summary of the studies that we examined is provided in Table 2, alongside the EPHPP rating and the number of subgroup analysis credibility criteria fulfilled for each.

**Table 2:** List of included studies evaluating public health interventions’ impact by SES.

*Lead author Date Country*	*Country*	*Intervention*	*Outcome measured*	*SES measure*	*EPHPP quality score*	*Subgroup analysis credibility score*
Batis et al. (2016)	Mexico	Taxation of foods and sugar sweetened beverages	Purchases of packaged foods	Education level and ownership of household assets	Weak	7
*Brownson et al. (1996)	USA	Health education; community based education	% change of the % of people who consume five portions of fruit and vegetables per day	Education level	Moderate	1
*Carcaise-Edinboro, McClish, Kracen, Bowen, and Fries (2008)	USA	Health education; Tailored feedback and self-help dietary intervention	Mean fruit and vegetable intake score	Education level	Strong	4
Colchero et al. (2016)	Mexico	Taxation of sugar sweetened beverages	Purchases of sugar sweetened beverages	Education level and ownership of household assets	Weak	4
*Connett and Stamler (1984)	USA	Dietary counselling intervention	Change in serum cholesterol (mg/dl)	Household income	Moderate	5
*Curtis, Adamson, and Mathers (2012)	UK	Health education: Cooking fair with cooking lessons accompanying personalised dietary goal settings	% change in mean food energy from fat	Area level index of multiple deprivation	Moderate	6
*Havas et al. (1998)	USA	Health education: healthy nutrition program aimed at adult women	Change in mean daily servings consumed of fruit and vegetables	Education level	Moderate	3
*Havas et al. (2003)	USA	Dietary counselling intervention	% change in fruit and vegetables consumed	Education level	Moderate	5
*Holme, Hjermann, Helgeland, and Leren (1985)	Norway	Dietary counselling intervention	% change in cholesterol	Social class	Moderate	3
*Hughes et al. (2012)	England	School based intervention	Change in portions of fruit and vegetables consumed	Area level index of multiple deprivation	Moderate	5
Jones, Taylor, Whittle, Evans, and Trotter (1997)	UK	Water fluoridation	Tooth decay	Area level index of multiple deprivation	Moderate	6
*Jouret et al. (2009)	France	Health education: healthy nutrition program aimed at children	Change in % of children overweight	Area level index of multiple deprivation	Strong	3
*Lowe, Horne, Tapper, Bowdery, and Egerton (2004)	UK	Health education: Healthy nutrition program aimed at children	% change in vegetables observed consumed	Free school meal entitlement	Weak	5
Nelson, Cooper, and Jackson (1995)	UK	Privatisation on employees of regional water authority	Employer job satisfaction and wellbeing	Occupation	Weak	5
*Plachta-Danielzik et al. (2007)	Germany	Health education: healthy nutrition programme aimed at children	Change in % prevalence of overweight	Parental education level	Weak	8
Vander Ploeg, Maximova, McGavock, Davis, and Veugelers (2014)	School-based physical activity programmes	10–11 year old school children	Physical activity levels	Household income and parental education level	Moderate	7
*Reynolds et al. (2000)	USA	Health education: healthy nutrition programme aimed at children	Portion of fruit and vegetables consumed	Household income	Moderate	3
*Smith, Owen, and Baghurst (1997)	Australia	Health education: healthy nutrition programme aimed at adults	Change in fat density consumed (g/4200 kcal)	Occupational prestige	Moderate	6
*Sorensen et al. (1998)	USA	Work based intervention	Change in geometric mean grams of fibre per 1000 kcals	Occupation	Moderate	9
*Toft, Jakobsen, Aadahl, Pisinger, and Jørgensen (2012)	Denmark	Dietary counselling intervention	Change in amount of fruit eaten by men (g/week)	Education level	Moderate	5
*Wendel-Vos et al. (2009)	Holland	Area based intervention	Difference in mean energy intake between intervention and control (MJ/d)	Education level	Strong	8

* Summary of intervention details and effects on health inequalities taken from McGill et al. (2015).

**Fig. 1:**
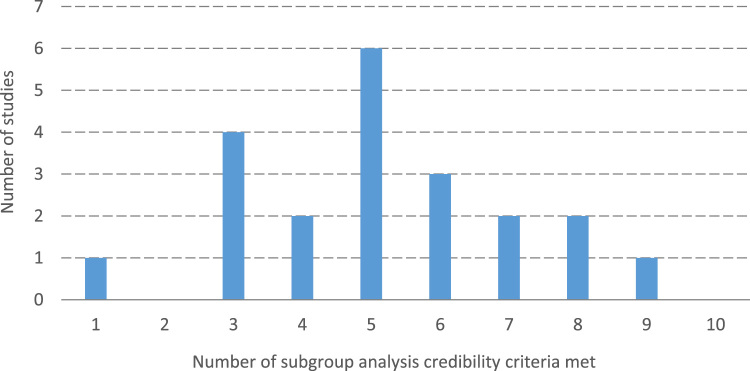
Frequency distribution of credibility of subgroup analysis scores amongst the included studies.

As shown in Table 2, 17 (81%) of the 21 studies that we scored were rated as being of either moderate or strong quality according to the EPHPP criteria. However, only 8 studies (38%) met at least 6 of the 10 criteria for credible subgroup analyses (Fig. 1).

Table 3 displays the number of studies that met each of the credibility criteria for subgroup analysis. The only criterion that was met by all of the studies was whether the subgroup variable (SES) was measured at baseline. SES was a stratification factor at randomisation in only 4 studies – although this criterion did not apply to 5 of the studies, which were not randomised trials, and so we adjusted the denominator of this criterion accordingly. Similarly, we note that the criterion “was the significant interaction effect independent, if there were multiple significant interactions?” did not apply to studies where a statistically significant interaction was not reported.

**Table 3:** Number and percentage of studies scoring positively on each of the credibility of subgroup analysis criteria.

Credibility of subgroup analysis criterion	Number (%) of studies
Is the subgroup variable a characteristic measured at baseline?	21/21 (100%)
Was the subgroup variable a stratification factor at randomisation?	4/17 (19%)*
Was the hypothesis specified a priori?	5/21 (24%)
Was the subgroup analysis one of a small number of subgroup analyses tested (≤5)?	10/21 (48%)
Was the test of interaction significant (interaction *p* < 0.05)?	17/21 (81%)
Was the significant interaction effect independent, if there were multiple significant interactions?	5/18 (24%)*
Was the direction of the subgroup effect correctly pre-specified?	4/21 (19%)
Was the subgroup effect consistent with evidence from previous studies?	19/21 (90%)
Was the subgroup effect consistent across related outcomes?	10/21 (48%)
Was there indirect evidence to support the apparent subgroups effect (biological rationale, laboratory tests, animal studies)?	13/21 (62%)

* Note: A lower denominator reflects the fact that these criteria were not applicable to all of the studies evaluated, either because the study was not an RCT or because the study did not report a significant interaction.

## 4 Discussion

The purpose of this study was to examine the credibility of subgroup analyses reported within a purposively sampled set of positively reviewed evaluations of diverse public health interventions, reporting differential effects by SES. Whilst the overall methodological quality of these studies was generally high - as evidenced by the positive ratings that the majority received on the EPHPP quality assessment tool - only 8 of the 21 studies that we examined met over half of the standard ten criteria for credible subgroup analyses. It is also important to note here that there is no particular recommended number of criteria that should be met before a subgroup analysis should be considered to be “credible”. Sun et al. (2010) argue against such dichotomous thinking, and instead suggest that the credibility of subgroup analyses should be assessed along a continuum running from “highly plausible” to “highly unlikely,” where researchers can be more confident that a reported subgroup effect is genuine as more of the credibility criteria are fulfilled.

Previous systematic reviews have found that the credibility of subgroup analyses reported in clinical trials is generally low (Saragiotto et al., 2016, Sun et al., 2012), although there has been very little review research that considered the credibility of such analyses in primary studies of public health intervention evaluations. Welch and colleagues (Welch et al., 2012) have previously reported a systematic “review of reviews” subgroup analyses across “PROGRESS-Plus” factors in systematic reviews of intervention evaluations. The PROGRESS-Plus acronym denotes sociodemographic characteristics where differential intervention effectiveness may be observed, and refers to: Place of residence; Race/ ethnicity/ culture; Occupation; Gender/sex; Religion; Education, and Social capital. The “Plus” further captures additional variables where inequalities may occur, such as sexual orientation. The scale and scope of Welch et al.’s research was different to ours - in part because the authors examined systematic reviews rather than primary studies, and because they considered a wider range of potential subgroup effects beyond SES, such those defined by gender and ethnicity. The authors nevertheless noted that only a minority of reviews even considered equity effects, and that, similar to our findings in the present study, the credibility of the analyses conducted within those reviews was rated by the authors as being relatively low. Specifically, only 7 of the 244 systematic reviews identified conducted subgroup analyses of pooled estimates across studies, and these analyses only met a median of 3 out of 7 criteria used by Welch et al. for credible subgroup analyses. Recent guidelines now emphasise the importance of following best practice guidance for planning, conducting and reporting subgroup analyses in equity-focused systematic reviews (Welch et al., 2012) – but, as our findings here demonstrate, this literature “has a long way to go” to comply with those guidelines. We note, in this regard, that a similar verdict has just been rendered by the authors of a new review of 29 systematic reviews of all types of public health interventions’ effects on health inequalities (Thomson et al., 2018).

Within our purposive sample of twenty-one *primary* evaluation studies of interventions, there was considerable variation in how many studies met each credibility criterion for subgroup analysis. One criterion that was met by relatively few of the studies that we examined were whether the subgroup effect was specified a priori, in terms of the subgroups examined. This is a crucial issue, as post-hoc analyses are more likely to yield spurious, false-positive subgroup effects (Sun, Ionnidis, Agoritsas, Alba, & Guyatt, 2014), and the results of such exploratory analyses are best understood as being hypothesis generating, rather than confirmatory (Burke, Sussman, Kent & Hayward, 2015; Oxman & Guyatt, 1992). A related criterion that few studies met was whether the direction of the effect was correctly pre-specified by the researchers. This is an important point because the plausibility of any observed effect is lowered when researchers previously predict only that there will be an effect without specifying its direction, or when the observed effect is in the opposite direction to that which was predicted (Sun et al., 2010, Sun et al., 2014). Notwithstanding the fact that previous studies on any question can clearly be wrong, it is important not to over-interpret effects when the direction was not correctly pre-specified.

Several conceptual frameworks have been developed that researchers can refer to when considering *how* an intervention might have differential effects according to SES. With regard to interventions for diet and obesity for example, Adams et al. (2016) argue that the degree of agency required of individuals to benefit from an intervention is a major determinant of its equity impacts: interventions that require a high degree of individual agency are likely to increase health inequalities, whilst interventions that require a low level of agency are likely to decrease inequalities. Drawing on such theoretical frameworks to consider the differential impacts of interventions, at the planning stages of intervention evaluations, would help to improve the credibility of subgroup analyses considerably.

There is also a need for researchers working on equity-focused systematic *reviews* to consider the credibility of subgroup analyses reported within primary intervention studies, and to weigh the conclusions that can be drawn from those studies accordingly. It is important to note here that the credibility of subgroup analyses is not currently included in some of the quality appraisal tools commonly applied in systematic reviews, such as the EPHPP. This explains the relatively high EPHPP scores of the 21 studies we reviewed, compared to their relatively low scores on the Saragiotti et al. scoring tool for subgroup analyses. We conclude that the fourteen-year-old EPHPP tool for quality-scoring in such reviews needs updating to reflect more recent methodological developments, especially in subgroup analysis based on interaction effects. The more recent “PRISMA” extension (Welch et al. 2016) represents a significant improvement in this regard.

### 4.1 Strengths, limitations and future research

The primary strengths of this research are the diversity of intervention evaluations considered, and the use of the most up-to-date and comprehensive set of criteria for credible subgroup analyses. The main limitation of this research is that the studies we examined were not identified via a systematic review of the literature, and this sample therefore cannot be considered to be representative of the field. In particular, the majority of the studies included were selected from a systematic review of interventions designed to promote healthy eating (McGill et al., 2015), although the range of policy and programme interventions evaluated in those studies was remarkably wide, spanning the full “degree of individual agency” typology laid out by Adams et al. It is therefore unclear whether these findings would generalise to the wider public health intervention literature, purporting to inform policy makers on “what works to reduce health inequalities by SES.” There is now a need to apply these credibility criteria to a fully representative set of evaluation studies of public health interventions.

In addition, the credibility criteria for subgroup analyses that we applied were originally designed to be applied to RCTs (Oxman and Guyatt, 1992, Sun et al., 2010), as is most clearly reflected by the criterion, “*was the subgroup variable a stratification factor at randomisation?*” More recent writings in the field of public health evaluation emphasise the role of sophisticated non-RCT quasi-experimental designs however, such as difference-in-differences with fixed effect variables for unidentified, non-time-varying confounders (Barr et al., 2016, Craig et al., 2011). The existing criteria for assessing the credibility of subgroup analyses may therefore need to be further adapted before being applied more widely to the public health intervention literature, where non-RCT designs are widely utilised.

The scope of this study was also limited to evaluation studies that reported differential intervention effects by SES. In this context, our interest was primarily in the likelihood that a Type I error is made, where false-positive subgroup effects are identified and reported. Equally important however is the possibility of Type II errors, where researchers erroneously do not find any evidence of differential effects by SES. Such errors may be relatively common, as evaluation studies that are designed to test the main effects of interventions will likely be under-powered to detect interaction effects between the treatment andpotential effect modifiers (Brookes et al., 2004).

Finally, in addition to evidence on the effectiveness of public health interventions, both researchers and policy makers have highlighted the need to identify the theoretical underpinnings of interventions, and to better understand the *causal pathways and mechanisms* through which interventions generate differential health outcomes by SES (Funnell & Rogers, 2011). However, we found that the primary studies we selected did not contain sufficient contextual and qualitative information to provide significant insights into those mechanisms. In this sense, the public health intervention literature we sampled presents another sort of evidence gap. That gapmakes the assessment of the *external* validity of any demonstrated effect on health inequalities particularly hard to judge, because inadequate theory and contextual detail are included in published evaluations to enable the reader to make an informed judgement about external validity of the results (Craig et al., 2008, Moore et al., 2015). As pointed out by Pawson (2006), the widespread adoption of newer forms of more qualitatively oriented, “realist” review would make an excellent counterpoint to purely quantitative assessments of effect-size per se. Realist review methods would allow more informed contextual interpretation and better identification of potential mechanisms of action of any given intervention, and their implications for a study’s external validity. We doubt that it would be helpful to merely issue more guidelines on such aspects of structured reviews of the equity aspects of public health interventions. We prefer the longer-term (and much slower) strategy of changing standard practice in this field so that future primary studies are simply expected by reviewers to provide richer contextual information. Such information would help to illuminate mechanisms of interventions’ effects, especially when they are differential across SES subgroups.

## 5 Conclusions

There is increasing interest amongst researchers and policy makers in identifying interventions that could potentially reduce (or increase) health inequalities by SES. The evidence regarding which interventions may be effective in doing so is often derived through subgroup analyses conducted in evaluation studies, which test whether the effect of the intervention differs according to participants’ SES. The methodological credibility of such analyses is only infrequently routinely considered, and our experience of applying established credibility criteria to a purposively selected set of evaluation studies suggests that this is an underappreciated problem. Researchers and consumers of the health inequalities literature should therefore make routine use of such criteria when weighing the evidence on which interventions may increase or reduce health inequalities.
